# Diagnostic dilemmas in a patient with multivascular embolic stroke

**DOI:** 10.1007/s12471-015-0720-7

**Published:** 2015-06-02

**Authors:** C.P.A. Delsing, M. van Duijnhoven, C. Arnoldussen, J. le Noble

**Affiliations:** 1 ICU, VieCuri Medical Centre, PO Box 1926, 5900 BX Venlo, The Netherlands; 2 Radiology department, VieCuri Medical Centre, PO Box 1926, 5900 BX Venlo, The Netherlands

**Keywords:** Paradoxical embolism, Patent foramen ovale, Pancreatic cancer, Cerebral ischaemic stroke

## Abstract

We describe a patient admitted to the intensive care unit with aphasia, which was due to an embolic ischaemic cerebral stroke associated with a previously unknown patent foramen ovale. Eventually, this finding during echocardiography led us to the diagnosis of pancreatic cancer. The thrombotic complications of pancreatic cancer, in combination with a large, patent foramen ovale, support the mechanism of a paradoxical embolism through the patent foramen ovale as the cause of cerebral ischaemic stroke.

## Introduction

Paraneoplastic neurological manifestations in cancer patients are common and may precede the diagnosis of malignancy. In particular, the incidence of stroke in patients with cancer is notably higher than in the general population. Ischaemic stroke results mainly from a state of hypercoagulability, atherosclerosis or nonbacterial thrombotic endocarditis. However, a first-ever stroke revealing an undiagnosed, underlying malignancy is a very rare event with a poor outcome [1].

A patent foramen ovale (PFO) is a common cardiac anomaly with a reported incidence of 17–35 %, based on autopsy studies. An unexplained stroke can be the initial presentation of a PFO. Some studies have suggested that unexplained strokes due to PFOs occur in 12–41 % of the population studied [2, 3]. To the best of our knowledge, this is a rare case report of a paradoxical embolism in a patient with pancreatic cancer.

**Fig. 1:**
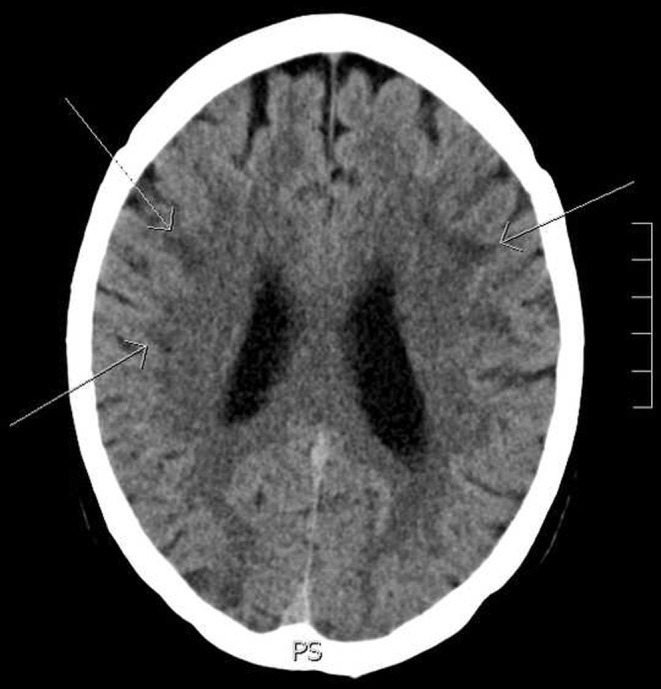
Computed tomography axial of the brain showing multiple hypodense subcortical areas suggestive of ischaemia

A 69-year-old woman was referred to our emergency department with a few days’ history of malaise, followed by acute aphasia. She had an unremarkable medical history, without any vascular risk factors. A neurological examination showed monoparesis of the right arm, aphasia and Babinski’s sign on the right side. The patient did not show any signs of endocarditis, and auscultation of the heart did not reveal any abnormalities. Laboratory analyses revealed slightly elevated inflammation parameters, hepatic dysfunction, leucocytosis and normocytic anaemia (aspartate aminotransferase, 87 (normal value, < 40) U/l; alanine transaminase, 90 (< 45) U/l; C-reactive protein, 103 (< 10) mg/l; haemoglobin, 6.8 (7.5–10.0) mmol/l; mean corpuscular volume, 83 (82–98) fl; leucocytes, 16.7 × 10^9^ (4.5–11.0)/l). The ECG showed a normal sinus rhythm without conduction abnormalities and without signs of pulmonary hypertension or left ventricular hypertrophy. Cerebral computed tomography (CT) revealed focal, hypodense lesions on multiple sites within different vascular territories (Fig. 1).

**Fig. 2:**
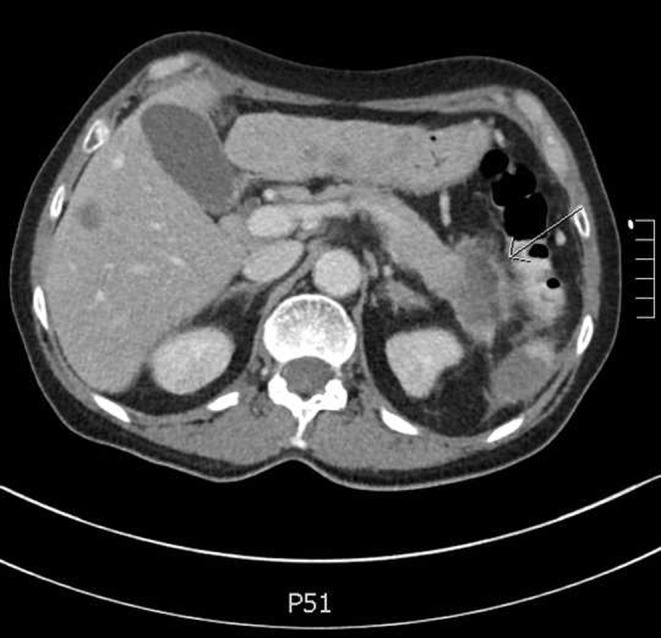
Computed tomography showing a pancreatic lesion, suspect for pancreatic cancer

On admission, the patient’s differential diagnosis included vasculitis, septic/embolic encephalopathy due to endocarditis or a paraneoplastic embolism. Vasculitis was unlikely due to negative tests for antineutrophil cytoplasmic antibody and antinuclear antibody. Transthoracic echocardiography (TTE) revealed an atrial septal defect, without any valve abnormalities. These observations, in combination with the cerebral imaging, allowed the final diagnosis of paraneoplastic embolism. Thoracic and abdominal CT identified the probable cause of the hypothesised paraneoplastic hypercoagulable state: pancreatic cancer (cauda) with liver and adrenal metastasis (Fig. 2). Because of the poor prognosis for this condition, further investigations were not conducted and palliative care was provided at home.

## Discussion

In this case report, we demonstrate that TTE is a valuable tool in the diagnosis of unexplained, embolic, ischaemic, cerebral stroke in a patient with a hypercoagulable state due to an unknown cancer. The incidence of hypercoagulability in patients with cancer varies between 1 and 11 % clinically, and by up to 50 % in autopsy series [4]. Arterial thromboembolisms are very rare, but hypercoagulability, induced by malignancy, is a cause of unexplained intravascular thrombosis. Under these conditions, endothelial cells may express cytokine-regulated procoagulant factors, and elevated platelet reactivity due to platelet–tumour interactions may also contribute to the hypercoagulable state [5]. In 1865, Trousseau was the first to describe migratory, superficial thrombophlebitis. He linked this hypercoagulable state with malignancies, usually mucin-producing adenocarcinomas and digestive cancers [6]. In pancreatic cancers, there is a particularly high risk of a tumour in the corpus/cauda, compared with in the caput [7].

The presence of a paradoxical embolism through a PFO, which has a reported prevalence of up to 35 % of the normal population, might be responsible for more arterial embolic events than is currently understood. In our patient, signs of arterial or venous thromboembolisms were not present elsewhere.

## Imaging

Screening for occult cancer in a patient with a first-ever stroke or thrombosis is not cost-effective; an active search for such cancer is only indicated in the subgroup of patients with a suspected malignancy [7]. However, echocardiography is essential in patients showing cerebral ischaemic stroke within different vascular territories. Echocardiography (sensitivity, 88 %; specificity, 97 %), initially coupled with lower-extremity venous Doppler ultrasound, is both simple and noninvasive, and should therefore be included in the diagnostic evaluation of a multivascular embolic stroke [8]. Unfortunately, due to the emergency setting, no proper data storage of the echocardiographic images was performed in our patient.

## Conclusions

This was a rare case of a patient who had signs and symptoms of a first-ever embolic stroke as the initial presentation of pancreatic cancer; TTE was essential in establishing the diagnosis. In our patient, the only finding that was linked with the embolic, ischaemic stroke was the large PFO. This, in combination with the hypothesised hypercoagulability due to malignancy, makes a paradoxical embolism the probable cause of the stroke.

If a patient presents with signs and symptoms of embolic ischaemic stroke, a PFO with paradoxical embolism should be considered. Present guidelines suggest TTE as an optional diagnostic tool. However, in patients with multivascular embolic stroke, TTE is essential in establishing the diagnosis and should therefore be a standard procedure. Also, in an emergency setting, proper data storage is mandatory.

### Funding

None.

### Conflict of interests

None declared.
